# Feasibility Study of Triple-low CCTA for Coronary Artery Disease Screening Combining Contrast Enhancement Boost and Deep Learning Reconstruction

**DOI:** 10.31083/RCM31334

**Published:** 2025-06-30

**Authors:** Zhihua Wu, Min Chen, Yingwen Wei, Chen Shen, Wen Han, Rulin Xu, Zhenyuan Zhou, Jiexiong Xu

**Affiliations:** ^1^Department of Radiology, The Fourth Affiliated Hospital, Guangzhou Medical University, 511300 Guangzhou, Guangdong, China; ^2^Research Collaboration, Canon Medical Systems, 510623 Guangzhou, Guangdong, China

**Keywords:** CE-Boost, deep learning reconstruction, coronary artery disease, coronary CTA, low radiation dose

## Abstract

**Background:**

The aim of this study was to compare the image quality of coronary computed tomography angiography (CCTA) images obtained using contrast enhancement boost (CE-boost) technology combined with deep learning reconstruction technology at a low dose and low contrast agent flow rate/dosage with traditional CCTA images, while exploring the potential application of this technology in early screening of coronary artery disease.

**Methods:**

From March 2024 to September 2024, 60 patients suspected of having coronary artery stenosis were enrolled in this study. Ultimately, 46 patients were included for analysis. Based on different acquisition protocols, divided into Group A and Group B. Group A underwent conventional computed tomography (CT) angiography with a tube voltage of 120 kV, a contrast agent injection rate of 6 mL/s, and a dosage of 0.9 mL/kg. Group B received a triple-low CCTA protocol with a tube voltage of 100 kV, a contrast agent injection rate of 2 mL/s, and a dosage of 0.3 mL/kg. Additionally, Group C was created by applying CE-Boost combined with a deep learning reconstruction technique to Group B images. The radiation dose and contrast agent dosage were compared between Group A and Group B. The image quality of the three groups, including CT values, background noise, signal-to-noise ratio (SNR), and contrast signal-to-noise ratio (CNR), was also compared, with *p* < 0.05 indicating significant statistical differences.

**Results:**

Our results indicate that Group A required 67.8% more contrast agent and a 52.0% higher radiation dose than Group B (64.68 ± 3.30 mL vs. 20.19 ± 2.22 mL, 6.21 (4.60, 7.78) mSv vs. 2.05 (1.42, 4.33) mSv, all *p* < 0.05). Image analysis revealed superior subjective scores in Groups A (4.68 ± 0.72) and C (4.38 ± 0.95) versus Group B (4.25 ± 0.10) (both *p* < 0.05), with no statistical difference between Groups A and C. CT values were significantly elevated in Group A across all vessels compared to both Groups B and C (*p* < 0.05), while Group C exceeded Group B post CE-Boost. SNR comparisons showed Group A dominance over B in the proximal right coronary artery (RCA-1)/left main coronary artery (LM)/left anterior descending coronary artery (LAD)/left circumflex coronary artery (LCX) and over C in the RCA-1/LM (*p* < 0.05), contrasting with the superiority of SNR in Group C versus B in the middle right coronary artery/distal right coronary artery (RCA-2/3)/LM/LAD/LCX. CNR analysis demonstrated an equivalent performance between A and C, though both groups significantly surpassed Group B (A vs. B: *p* < 0.05; C vs. B: *p* < 0.05).

**Conclusion:**

The triple-low CCTA protocol using CE-Boost technology combined with deep learning reconstruction, achieved a 52% reduction in radiation exposure and a 67.8% reduction in contrast agent usage, while preserving diagnostic image quality (with CNR and noise levels comparable to standard protocols). This demonstrates its clinical feasibility for repeated coronary evaluations without compromising diagnostic accuracy.

## 1. Introduction

Coronary artery disease (CAD) is the leading cause of death worldwide [1]. CAD usually manifests varying degrees of coronary stenosis; with critical lesions potentially inducing hemodynamically significant flow reduction that may progress to myocardial ischemia or life-threatening complications [2]. Early diagnosis in suspected CAD patients is crucial for improving their prognosis and reducing complications [3]. Currently, coronary angiography (CAG) is the gold standard for diagnosing coronary artery occlusion [4], though it remains an invasive procedure [5]. Coronary computed tomography angiography (CCTA), as a non-invasive imaging modality, has gradually been used as a reliable alternative and is widely utilized in the diagnosis and screening of CAD [6]. However, it should be noted that although CCTA has a reduced radiation dose and contrast agent dosage compared to CAG, it still requires a certain amount of radiation and iodinated contrast agent to ensure adequate image quality. High radiation doses are often associated with carcinogenic potential [7]. Additionally, higher volumes of iodinated contrast agents may increase the risk of contrast-induced nephropathy (CIN) [8]. In the PROTECTION VI study [9], CCTAs of 4006 patients from 61 international study sites were analyzed, and the results showed that a low dose of contrast agent led to decreased intravascular computed tomography (CT) values, and the extent of the decrease was correlated with the patient’s body mass index (BMI). Therefore, there is an urgent need for innovative technical solutions to improve image quality while effectively reducing radiation dose and burdens.

Contrast-enhancement-boost (CE-boost) is a CT postprocessing technique that utilizes subtraction techniques and registration algorithms to mitigate motion artifacts between different phases, ensuring precise registration [10]. CE-boost enhances vascular opacification of blood vessels and reduces the amount of contrast required during angiography by subtracting the enhanced image from the pre-enhanced image [11]. Currently, CE-boost technology has effectively reduced the contrast agent dosage for vascular angiography in the carotid arteries [12], portal veins [13], and in abdominal vascular imaging [11]. Additionally, we employed Advanced Intelligent Clear IQ Engine (AiCE) technology to address the issue of increased image noise due to a reduced radiation dose. This deep learning-based reconstruction (DLR) technique utilizes deep convolutional neural networks (DCNN) to distinguish anatomical structures from quantum noise in medical images [14]. Consequently, reducing noise from the signal and producing high-quality images while reducing radiation dose [15]. Compared with iterative reconstruction, AiCE can reduce the dose of radiation by up to 40% in CCTA, improving image quality [16]. However, research remains scarce regarding the image quality of low-dose radiation and low contrast agent flow rate/dosage in CCTA using CE-boost technology combined with AiCE reconstruction.

Conventional CCTA protocols, while non-invasive, require substantial radiation doses (6–10 mSv) and contrast agent volumes (60–100 mL), which pose risks of renal impairment and cumulative radiation exposure [7, 8]. Triple-low CCTA (low kV, low contrast flow rate, low contrast dose) addresses these limitations but may result in reduced vascular enhancement and increased noise. The aim of this study was to compare the image quality of CCTA images acquired with triple-low protocol using CE-boost technology combined with AiCE reconstruction technology, with conventional CCTA images. We hypothesized that CE-Boost can effectively enhance the contrast of coronary artery imaging, while AiCE reconstruction can effectively preserve the image quality of CCTA. The integration of CE-Boost and AiCE makes the triple-low CCTA protocol feasible.

## 2. Methods

### 2.1 Participants

**Fig. 1.**
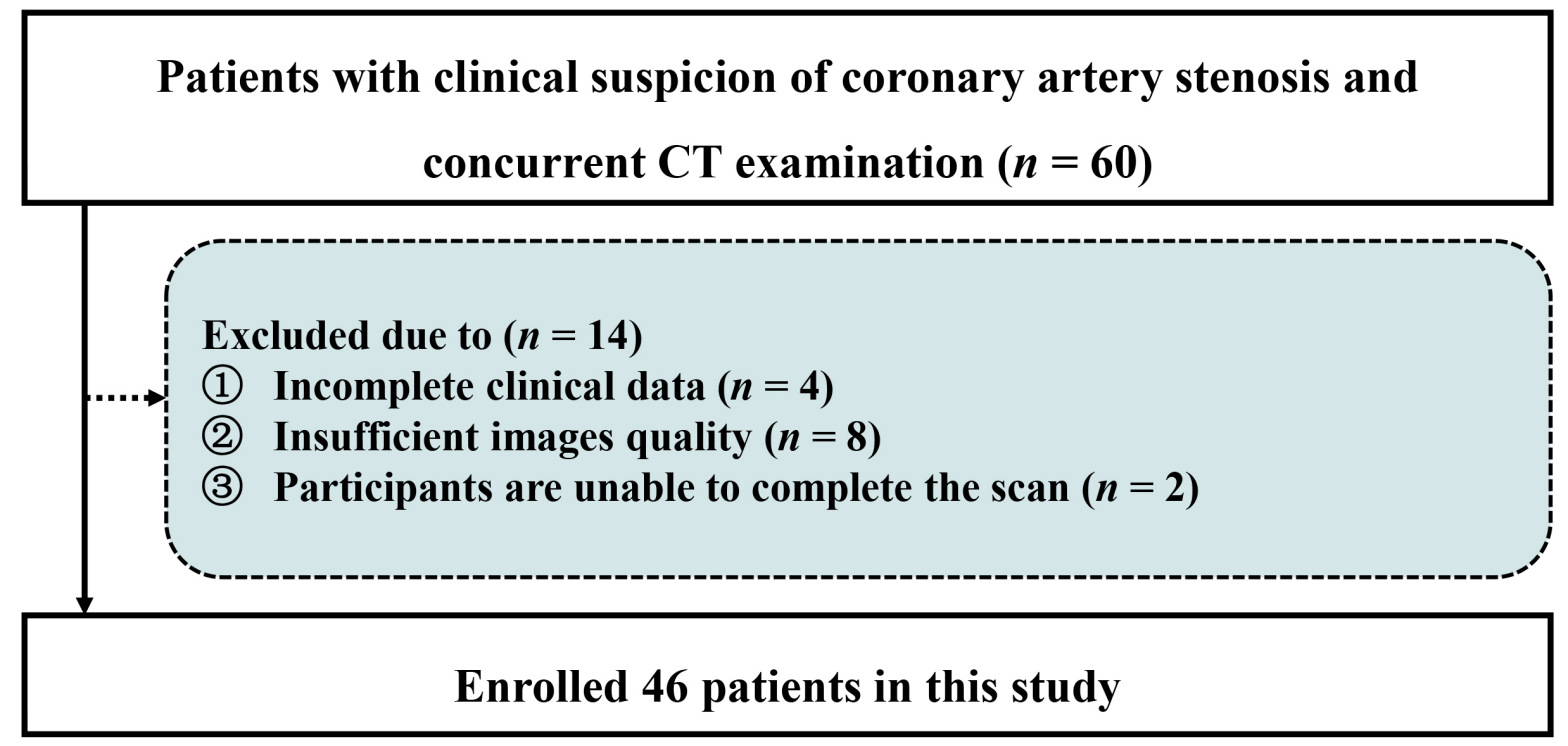
**Patient enrollment workflow**. CT, computed tomography.

This study prospectively included 60 patients with suspected coronary artery stenosis from March 2024 to September 2024. The Ethics Committee of our hospital approved this prospective study (2024-H-003), and all participants provided written informed consent prior to undergoing a CT scan. Inclusion criteria were as follows: (a) no history of contrast agent allergy; (b) normal renal function (glomerular filtration rate ≥80 mL/min); (c) no prior history of cardiac surgery. Exclusion criteria were as follows: (a) incomplete clinical data; (b) respiratory and motion artifacts resulting in insufficient images for reconstruction; (c) scan interruption due to procedural discomfort. The participant flowchart is depicted in Fig. 1. Clinical characteristics were recorded, including patient age, gender, heart rate during examination, weight, height, and calculated BMI. Of 60 enrolled patients, 14 were excluded: 8 due to motion artifacts, 4 with incomplete clinical data, and 2 for scan interruptions. The final analysis included 46 patients (Group A: 25, Group B/C: 21).

### 2.2 Scanning Protocol

All CT angiography examinations were performed on a 320-row CT scanner (Aquilion ONE Genesis, Canon Medical System, Otawara-shi, Japan). The CT scanner underwent daily quality assurance tests using air calibration. Tube current modulation and detector sensitivity were recalibrated semi-annually using a water phantom. Water phantom calibration ensured the measurement of CT number accuracy, noise, and uniformity. Participants were positioned supine with their heads oriented forward, with their hands crossed to support their heads, and participated in breathing training. Scans were performed after inhaling and holding their breath as instructed. The scanning range extended from the bifurcation of the trachea to the diaphragmatic surface of the heart, using cardiac volume scanning with a spiral time of 0.275 seconds and a collimator width of 160 mm. All patients were divided into Group A and Group B according to different acquisition protocols.

Group A: Tube voltage: 120 kV, automatic milliampere-second technology. The contrast agent employed was a concentration of 370 mgI/ mL (iodoprolol injection, Bayer Healthcare, Berlin, Germany), injected via a high-pressure injector using the Bayer MEDRAD Stellant CT Injection System (Stellant D-CE, Pittsburgh, PA, USA) through the median cubital vein. Contrast agent was delivered at 6 mL/s, with a dosage of 0.9 mL/kg [17, 18]. After the contrast agent injection was completed, 30 mL of physiological saline was flushed at the same rate. The CCTA scan was triggered once the detection threshold of the descending aorta reached 150 Hounsfield Unit (HU).

Group B: Tube voltage: 100 kV. Contrast agent was delivered at 2 mL/s, with a dosage of 0.3 mL/kg. The rest of the scanning parameters were consistent with those of Group A.

### 2.3 Image Reconstruction and Post-processing

Images from both Group A and Group B were reconstructed using hybrid iterative reconstruction (AIDR 3D, FC43 kernel, Canon Medical Systems, Otawara-shi, Japan) for all transverse CT images, with a slice thickness of 0.5 mm and intervals of 0.25 mm. The original data for Group C images were derived from Group B. Group C images were generated from the raw data of Group B: the raw data were reconstructed using deep learning reconstruction (AiCE, Cardiac Standard, Canon Medical Systems, Otawara-shi, Japan) to obtain the CT images, with a slice thickness of 0.5 mm and intervals of 0.25 mm. Further post-processing was performed using CE-Boost (SURESubtraction Iodine Mapping, Canon Medical Systems, Otawara-shi, Japan) to generate the final Group C images.

### 2.4 Measurement of Radiation Exposure

To estimate radiation dose exposure, we recorded the dose length product (DLP) and volume CT dose index (CTDI_vol_) for all participants. According to international recommendations, the effective dose (ED) was calculated by multiplying the DLP by an adult chest-specific conversion factor (0.014 mSv Gy⁻^1^⋅cm⁻^1^) [19].

### 2.5 Subjective Evaluation

The image quality of the three sets of images was evaluated by two radiologists with 8 and 6 years (CS and WH) of experience in coronary artery computed tomography angiography (CTA) diagnosis, respectively. Both radiologists were blinded to the patients’ clinical information and image processing methods. They used a 5-point scoring system to evaluate the clarity, contrast, fine structure, and image noise of the original and post-processed images. A visual reference for the scoring criteria is provided in **Supplementary Fig. 1**, The scoring criteria were as follows [15]:

5 points: Excellent image contrast, no obvious noise, clear display of fine structures with sharp boundaries, and clear visualization of the entire process and terminal segments of vascular reconstruction.

4 points: Good image contrast, increased noise, clear display of fine structures with well-defined boundaries, and fewer peripheral branches throughout vascular reconstruction.

3 points: Fair image contrast, moderate noise, clear vascular reconstruction, and slightly unclear display of small anatomical structures.

2 points: Poor image contrast, significant noise, unclear display of small anatomical structures, with difficulty in identification, and unclear visualization of vascular reconstruction.

1 point: Very poor image contrast, excessive noise, inability to distinguish small anatomical structures, and unclear display of vascular reconstruction.

An image score of ≥3 was considered to meet clinical diagnostic requirements, while a score of ≤2 was considered not to meet these requirements.

### 2.6 Objective Evaluation

Two experienced radiologists (MC and YW), with 15 and 9 years of experience in coronary artery CTA diagnosis, respectively, evaluated the image quality of three types of images without knowing the patients’ clinical information or image processing methods.

Regions of interest (ROI) were delineated in the left main coronary artery (LM), left anterior descending coronary artery (LAD), left circumflex coronary artery (LCX), and the tertiary branches of the right coronary artery (proximal right coronary artery (RCA-1), middle right coronary artery (RCA-2), and distal right coronary artery (RCA-3)). The ROI was placed in the center of the blood vessel, avoiding the vessel wall, plaque, calcification, and stents. The ROI area for RCA-1 and LM was about 15 mm^2^, RCA-2 and LAD about 10 mm^2^, and RCA-3 and LCX about 5 mm^2^. An ROI with an area of 50 mm^2^ was selected in the right erector spinae muscle (RES), and the resulting standard deviation (SD) was considered the background SD.

CT values and noise SD of these blood vessels were recorded, and the signal-to-noise ratio (SNR) (1) and contrast-to-noise ratio (CNR) (2) were calculated [20]. The calculation formulas were as follows:

(1) $$S\,N\,R=\frac{H\,U_{C\,A}}{S\,D}$$

(2) $$C\,N\,R=\frac{H\,U_{C\,A}-H\,U_{R\,E\,S}}{\sqrt{\frac{S\,D_{C\,A}}{S\,D_{R\,E\,S}}}}$$

In the above formulas, HU_C⁢A_ represents the signal intensity of the coronary artery, HU_R⁢E⁢S_ represents the signal intensity of the erector spinae muscle, SD_C⁢A_ represents the standard deviation of the coronary artery, SD represents local noise, and SD_R⁢E⁢S_ represents the standard deviation of the erector spinae muscle.

### 2.7 Statistic Analysis

We used SPSS 26.0 (IBM Corporation, Armonk, NY, USA) and R 4.2.1 (The R Foundation for Statistical Computing, Vienna, Austria) for data analysis and processing and performed a normality test on the obtained data. Data conforming to a normal distribution were reported as mean ± SD ($\bar{x}$ ± s). Independent samples *t*-tests were used for comparisons between two groups, while one-way ANOVA was used for comparisons among multiple groups. Data not following a normal distribution were represented by the median and interquartile range. The Mann-Whitney U test was used for comparisons between two groups, and the Bonferroni correction was used for comparisons among multiple groups. Categorical variables were compared using the chi-square test. A *p* value of <0.05 was considered statistically significant. The consistency of CT values, noise measurements, and subjective scores was analyzed using intraclass correlation coefficients (ICCs), which were classified as poor (ICC <0.50), moderate (ICC 0.50–0.74), good (ICC 0.75–0.89), or excellent (ICC >0.90). A priori power analysis (G*Power 3.1, Heinrich-Heine-Universität Düsseldorf, Düsseldorf, Germany) indicated 17 patients per group were required (α = 0.05, power = 0.8, effect size d = 1.2). Our study included 21 participants in Group B/C, exceeding this threshold. Intergroup comparisons were performed using one-way ANOVA with Bonferroni correction for multiple comparisons. All statistical tests reported adjusted *p*-values (*p*_adj_), mean differences with 95% confidence intervals, and effect sizes calculated via Cohen’s *d* method.

Additionally, we performed age and BMI adjusted statistical analyses to account for potential confounders. The results of these analyses, including the adjusted *p*-values for the comparisons between Groups A and B and between Groups A and C. For the comparison between Groups B and C, no adjustment was necessary as they belong to the same patient cohort.

## 3. Results

### 3.1 Basic Information About Patients

This study included a total of 46 participants (29 males and 17 females). Group A consisted of 25 participants (19 males and 6 females) with an average age of 57.76 ± 10.62 years. Group B consisted of 21 participants (10 males and 11 females) with an average age of 62.05 ± 11.52 years.

**Fig. 2.**
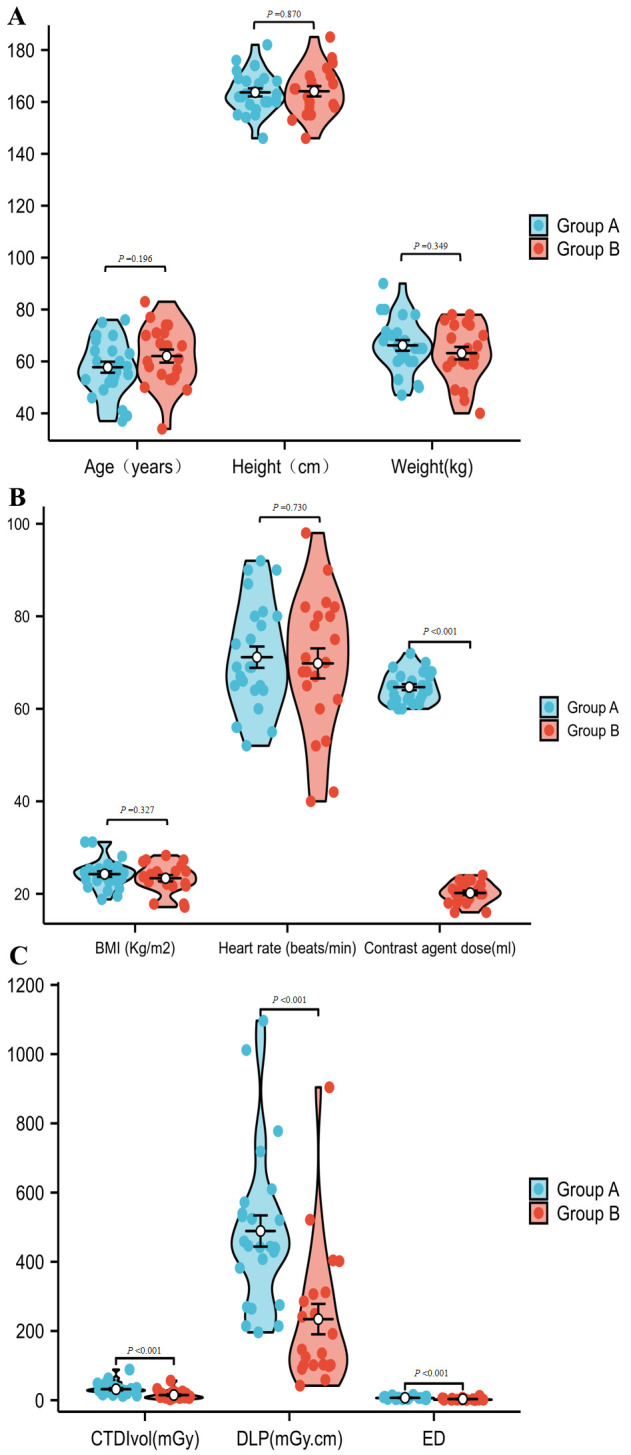
**Comparison of patient demographics, contrast agent dosage, and radiation dose metrics between Group A (conventional protocol) and Group B (triple-low protocol)**. (A,B) Comparison of general patient data statistics and dosage of contrast agents. (C) Comparison of patient radiation dose metrics. BMI, body mass index; CTDI_vol_, CT dose index; DLP, dose length product; ED, effective dose.

We compared the clinical data and radiation exposure of the patients and found that the contrast agent dosage in Group A was significantly higher than those in Group B. However, there was no statistically significant difference between Group A and Group B in terms of age, gender, heart rate during examination, height, weight, BMI, history of smoking, hyperlipidemia, diabetes, family history of coronary heart disease and hypertension (all *p* > 0.05). Detailed data can be found in Table 1, Fig. 2A–C.

**Table 1.** **Clinicopathologic characteristics of participants**.

Characteristic		Group A (n = 25)	Group B (n = 21)	χ ^2^ */Z/T*	*p* value
Age (mean ± standard deviation, years)		57.76 ± 10.62	62.05 ± 11.52	–1.312	0.196^Δ^
Height (cm)		163.68 ± 7.78	164.10 ± 9.09	–0.167	0.870^Δ^
Weight (kg)		66.17 ± 10.25	63.19 ± 11.08	0.946	0.349^Δ^
BMI (kg/m^2^)		24.27 ± 3.00	23.37 ± 3.13	0.939	0.327^Δ^
Heart rate (beats/min)		71.16 ± 11.51	69.81 ± 14.89	0.347	0.730^Δ^
History of smoking (%)				0.002	0.963^◊^
	No	19 (76.0)	17 (81.0)		
	Yes	6 (24.0)	4 (19.0)		
Hyperlipidemia (%)				0.014	0.905^◊^
	No	20 (80.0)	18 (85.7)		
	Yes	5 (20.0)	3 (14.3)		
Diabetes (%)				0.206	0.650^◊^
	No	19 (76.0)	18 (85.7)		
	Yes	6 (24.0)	3 (14.3)		
Family history of coronary heart disease (%)				0.117	0.732^◊^
	No	22 (88.0)	20 (95.2)		
	Yes	3 (12.0)	1 (4.8)		
Hypertension (%)				0.636	0.425^◊^
	No	9 (36.0)	10 (47.6)		
	Yes	16 (64.0)	11 (52.4)		
Contrast agent dose (mean ± standard deviation, mL)		64.68 ± 3.30	20.19 ± 2.22	52.496	<0.05^Δ^
CTDI_vol_ (median and interquartile range, mGy)		31.788 (21.750, 34.700)	14.914 (6.850, 19.350)	–4.179	<0.05^●^
DLP (median and interquartile range, mGy·cm)		443.70 (328.55, 555.75)	149.40 (101.15, 309.15)	77.00	<0.05^●^
ED (median and interquartile range, mSv)		6.21 (4.60, 7.78)	2.05 (1.42, 4.33)	–4.091	<0.05^●^

Numbers in parentheses were presented as percentages. ^●^: Continuous variables were compared by using the Mann-Whitney U test. ^◊^: Categorical variables were compared by using the chi-square test. ^Δ^: Continuous variables were compared using independent-sample *t* test. A significant difference was considered when the *p* value < 0.05.

### 3.2 Images Quality Assessment

#### 3.2.1 Subjective Evaluation

**Fig. 3.**
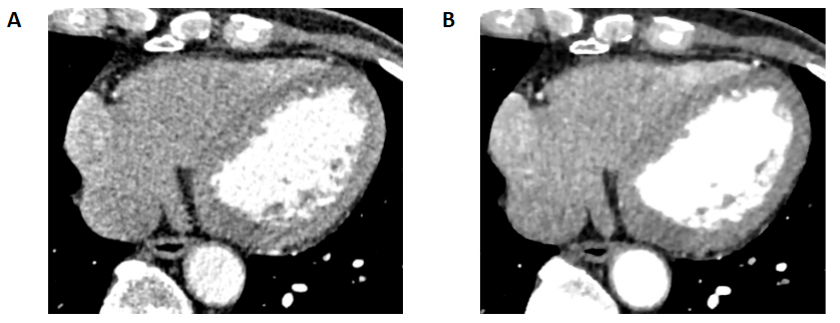
**Comparison of AiCE effects: Patient is male, 71 years old**. (A) (Score 3: moderate noise) shows the patient’s RCA, LAD, and LCX vessels with blurred edges and unclear luminal visualization. (B) (Score 4: reduced noise, sharper lumen) shows the patient’s RCA, LAD, and LCX vessels with sharp edges and a clearer lumen after AiCE reconstruction compared to (A). AiCE, Advanced Intelligent Clear IQ Engine; RCA, right coronary artery; LAD, left anterior descending coronary artery; LCX, left circumflex coronary artery.

We assessed the image quality of the three groups, and the scores from the two doctors showed good consistency (ICC = 0.913). The average score of the two radiologists was used as the final score. We then calculated the mean subjective score and SD for each group of images: Group A: 4.68 ± 0.72, Group B: 4.25 ± 0.10 and Group C: 4.38 ± 0.95. The scores for Group A and Group C were significantly higher than those for Group B (both *p* < 0.001), with a representative CE-Boost comparison between Groups B and C provided in Fig. 3. There was no statistical difference between Group A and Group C.

#### 3.2.2 Objective Evaluation

**Fig. 4.**
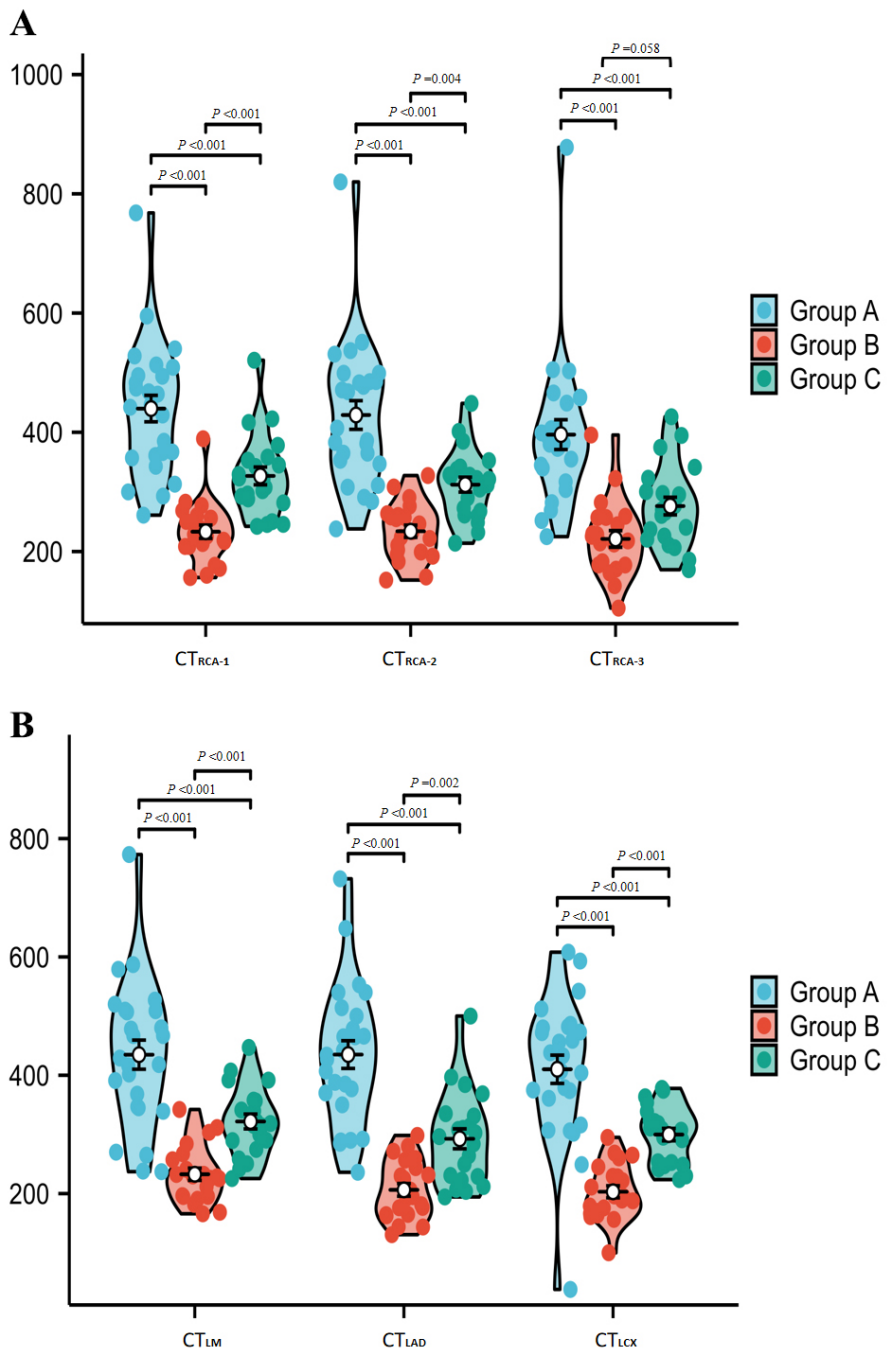
**Comparison of CT values of various vessels in the patient’s CCTA images**. (A) (RCA-1/2/3) and (B) (LM/LAD/LCX) display CT value measurements. In comparing intravascular CT values among the three groups, ​Group A demonstrated significantly higher CT values than both Groups B and C in all measured coronary segments (*p* < 0.05). Furthermore, Group C exhibited significantly higher CT values following CE-Boost application compared to Group B (*p* < 0.05). CCTA, coronary computed tomography angiography.

The measurements showed good consistency between the two doctors (ICC = 0.515~0.995). We analyzed the interobserver consistency of CT values and noise of each blood vessel in two groups of patients. The results showed that, RCA-1 (CT value ICC = 0.982, SD ICC = 0.59); RCA-2 (CT value ICC = 0.995, SD ICC = 0.964); RCA-3 (CT value ICC = 0.992, SD ICC = 0.810); LM (CT value ICC = 0.986, SD ICC = 0.515); LAD (CT value ICC = 0.973, SD ICC = 0.673); and LCX (CT value ICC = 0.976, SD ICC = 0.849). In comparing intravascular CT values among the three groups, Group A had significantly higher CT values than both Group B and Group C in all measured blood vessels (*p* < 0.05). Additionally, Group C had significantly higher CT values after performing CE-Boost compared to Group B (*p* < 0.05) (Table 2. Fig. 4A,B).

**Table 2.** **Comparison of image parameters among Group A, Group B, and Group C**.

Image parameters		Group A (n = 25)	Group B (n = 21)	Group C (n = 21)	*H/F*	*p* value	*p *^1^ value	*p *^2^ value	*p *^3^ value
CT value	CT_LM_	434.76 ± 123.05	232.65 ± 47.65	321.83 ± 57.30	31.861	<0.001*	<0.001*	<0.001*	<0.001*
	CT_LAD_	434.92 ± 117.25	206.26 ± 49.07	292.58 ± 77.25	39.725	<0.001*	<0.001*	<0.001*	0.002
	CT_LCX_	410.08 ± 119.41	203.01 ± 46.79	299.71 ± 47.94	36.522	<0.001*	<0.001*	<0.001*	<0.001*
	CT_RCA-1_	439.76 ± 110.99	233.22 ± 51.09	326.83 ± 68.07	35.729	<0.001*	<0.001*	<0.001*	<0.001*
	CT_RCA-2_	429.00 ± 120.24	234.01 ± 46.88	312.19 ± 57.16	31.214	<0.001*	<0.001*	<0.001*	0.004
	CT_RCA-3_	396.12 ± 124.88	221.19 ± 63.94	276.29 ± 67.66	21.754	<0.001*	<0.001*	<0.001*	0.058
SD	SD_LM_	25.23 (19.29, 30.30)	24.45 (18.87, 29.83)	23.71 (19.30, 29.52)	0.187	0.911	0.716	0.974	0.697
	SD_LAD_	28.85 (18.72, 38.31)	26.43 (19.70, 33.37)	23.46 (20.81, 31.47)	3.783	0.151	0.120	0.079	0.930
	SD_LCX_	26.53 (20.65, 36.82)	27.05 (21.01, 32.90)	28.66 (20.90, 38.46)	0.343	0.842	0.834	0.683	0.538
	SD_RCA-1_	29.22 (22.31, 35.00)	29.54 (26.20, 31.43)	26.97 (18.95, 40.12)	0.158	0.924	0.886	0.766	0.697
	SD_RCA-2_	32.50 (21.17, 58.25)	26.89 (18.39, 35.42)	27.80 (24.68, 32.59)	14.599	<0.001*	0.003*	0.001*	0.399
	SD_RCA-3_	29.23 (22.19, 53.25)	23.36 (19.91, 29.24)	22.77 (19.30, 33.03)	19.313	<0.001*	<0.001*	<0.001*	0.792
SNR	SNR_LM_	18.11 ± 6.56	10.25 ± 3.63	13.98 ± 5.30	12.232	<0.001*	<0.001*	0.012	0.028
	SNR_LAD_	15.09 ± 8.20	8.27 ± 2.74	12.85 ± 6.28	6.781	0.002	0.001	0.237	0.001
	SNR_LCX_	15.40 ± 8.27	7.51 ± 3.21	12.91 ± 8.03	7.431	0.001	<0.001*	0.234	<0.001*
	SNR_RCA-1_	17.71 ± 7.20	8.83 ± 2.98	12.00 ± 4.37	16.647	<0.001*	<0.001*	0.001	0.058
	SNR_RCA-2_	10.03 ± 4.71	8.60 ± 3.15	14.64 ± 8.94	5.800	0.005	0.429	0.012	0.002
	SNR_RCA-3_	9.43 ± 4.60	9.24 ± 3.22	11.96 ± 3.49	3.309	0.043	0.863	0.031	0.026
CNR	CNR_LM_	14.59 ± 4.50	7.27 ± 2.72	14.66 ± 4.61	23.572	<0.001*	<0.001*	0.956	<0.001*
	CNR_LAD_	16.70 ± 4.68	6.30 ± 2.60	13.16 ± 5.08	23.961	<0.001*	<0.001*	0.229	<0.001*
	CNR_LCX_	13.72 ± 5.17	6.11 ± 2.11	13.57 ± 4.42	23.356	<0.001*	<0.001*	0.904	<0.001*
	CNR_RCA-1_	14.90 ± 4.31	7.29 ± 2.95	15.06 ± 5.38	22.713	<0.001*	<0.001*	0.898	<0.001*
	CNR_RCA-2_	14.51 ± 4.76	7.29 ± 2.61	14.34 ± 5.48	18.375	<0.001*	<0.001*	0.897	<0.001*
	CNR_RCA-3_	13.07 ± 4.14	6.95 ± 3.45	12.39 ± 4.81	14.172	<0.001*	<0.001*	0.581	<0.001*

SNR, signal-to-noise ratio; CNR, contrast-to-noise ratio; (CT_LM_, SD_LM_, SNR_LM_, SNR_LM_), CT values, noise, signal-to-noise ratio, and contrast signal-to-noise ratio of the left main coronary artery; (CT_LAD_, SD_LAD_, SNR_LAD_, CNR_LAD_), CT values, noise, signal-to-noise ratio, and contrast signal-to-noise ratio of the left anterior descending coronary artery; (CT_LCX_, SD_LCX_, SNR_LCX_, CNR_LCX_), CT values, noise, signal-to-noise ratio, and contrast signal-to-noise ratio of left coronary artery circumflex branch; (CT_RCA-1_, SD_RCA-1_, SNR_RCA-1_, CNR_RCA-1_), CT values, noise, signal-to-noise ratio, and contrast signal-to-noise ratio of the proximal right coronary artery; (CT_RCA-2_, SD_RCA-2_, SNR_RCA-2_, CNR_RCA-2_), CT values, noise, signal-to-noise ratio, and contrast signal-to-noise ratio of the middle segment of the right coronary artery; (CT_RCA-3_, SD_RCA-3_, SNR_RCA-3_, CNR_RCA-3_), CT values, noise, signal-to-noise ratio, and contrast signal-to-noise ratio of the distal segment of the right coronary artery; *p *^1^, Group A vs. Group B; *p *^2^, Group A vs. Group C; *p *^3^, Group B vs. Group C. The *p* value was calculated using a one-way ANOVA test with Bonferroni correction, and a *p* value < 0.05* was considered statistically significant.

**Fig. 5.**
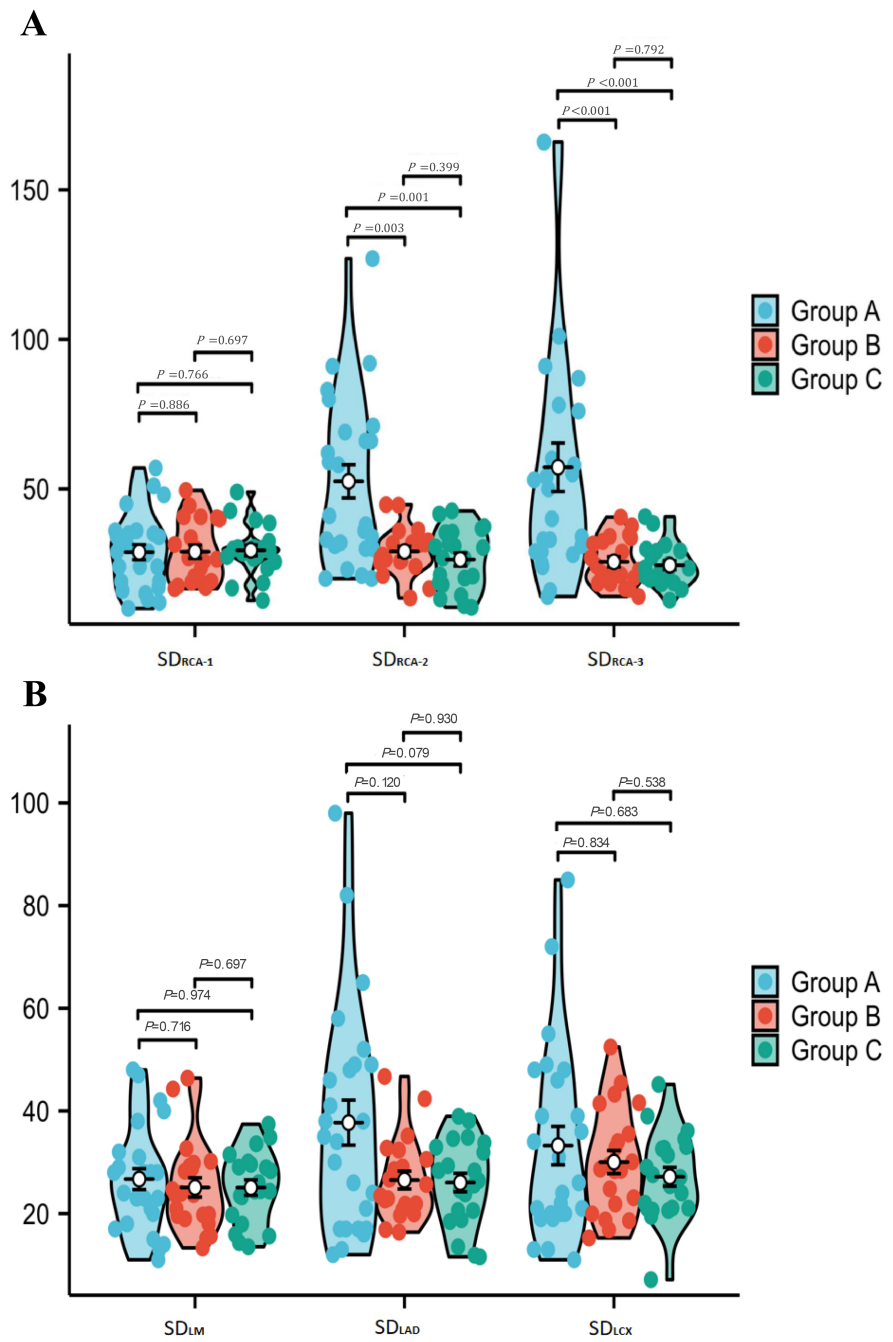
**Comparison of noise levels in various vessels of the patient’s CCTA images**. (A) (RCA-1/2/3) and (B) (LM/LAD/LCX) display noise level measurements. In comparing noise levels among the three groups, Group A demonstrated significantly higher noise levels than both Groups B and C in RCA-2 and RCA-3 (*p* < 0.05). No significant differences were observed in the remaining vessels (LM, LAD, LCX, or RCA-1). Furthermore, no statistical differences in noise levels were found between Group B and Group C in any vascular segments.

When comparing noise levels, Group A had significantly higher noise levels than both Group B and Group C in RCA-2 and RCA-3 (*p* < 0.05). No significant differences were found between Group A and Groups B and C in the remaining vessels, and there were no statistical differences in noise levels between Group B and Group C in any vessels all vascular segments (Table 2. Fig. 5A,B).

Group A had significantly higher SNRs than Group B in RCA-1, LM, LAD and LCX (*p* < 0.05), but no significant differences in other vessels (all *p* > 0.05). Group A show higher SNRs in RCA-1 and LM compared to Group C (*p* < 0.05); while Group C had higher SNRs in RCA-2 and RCA-3 (*p* < 0.05). There were no significant differences in the SNR for LAD and LCX between Groups A and C (*p* > 0.05).

**Fig. 6.**
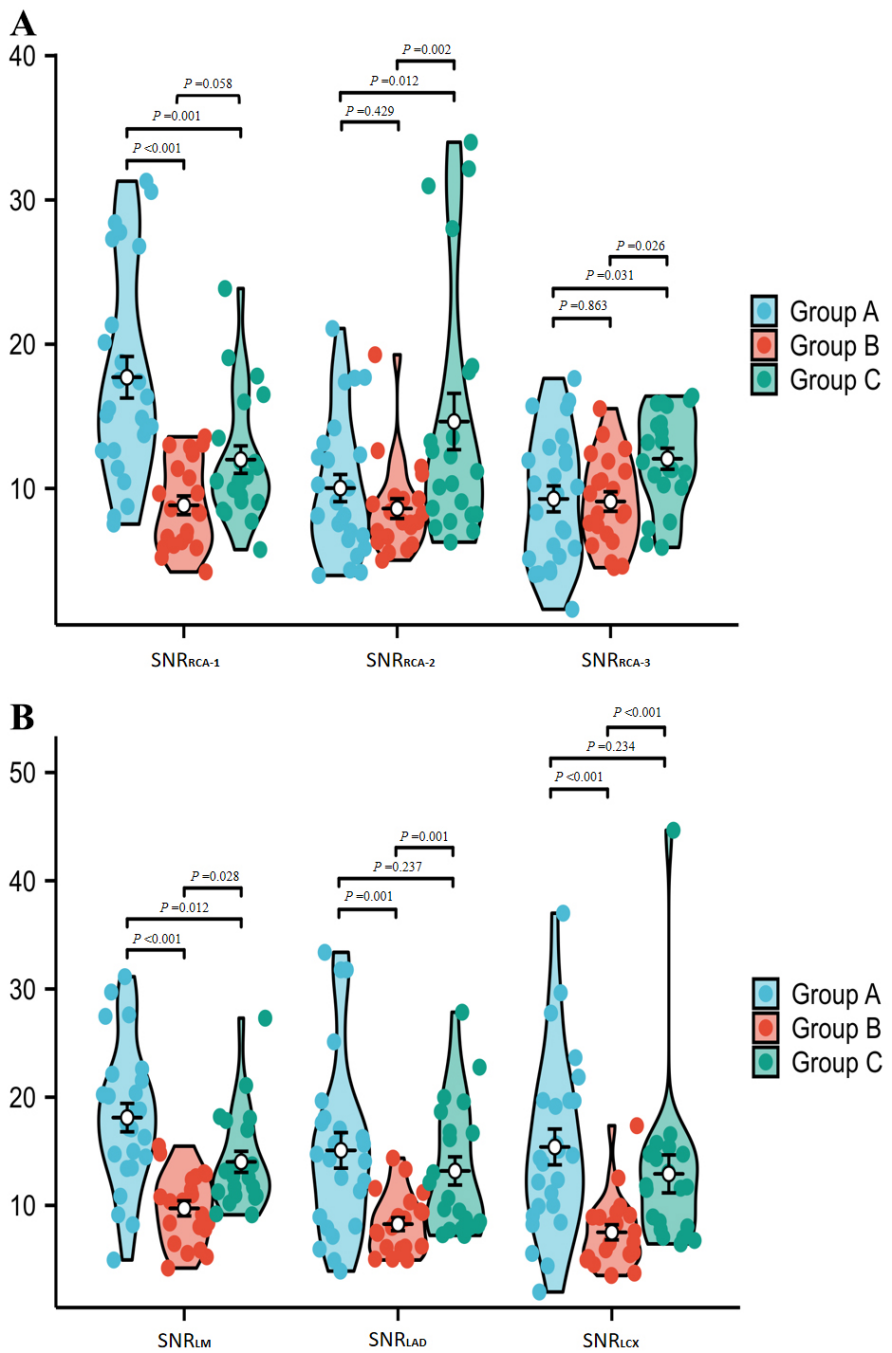
**Comparison of SNR in various vessels of the patient’s CCTA images**. (A) (RCA-1/2/3) and (B) (LM/LAD/LCX) display SNR measurements. Regarding SNR evaluations, Group A exhibited significantly higher SNRs than Group B in RCA-1, LM, LAD, and LCX (*p* < 0.05). Additionally, Group A had higher SNRs than Group C in RCA-1 and LM (*p* < 0.05). Group C demonstrated significantly greater SNRs than Group B in RCA-2, RCA-3, LM, LAD, and LCX (*p* < 0.05), with no significant difference in RCA-1.

When comparing Group B and Group C, Group C had substantially greater SNRs in RCA-2, RCA-3, LM, LAD, and LCX (*p* < 0.05), with no significant difference in RCA-1 (*p* > 0.05) (Table 2. Fig. 6A,B).

**Fig. 7.**
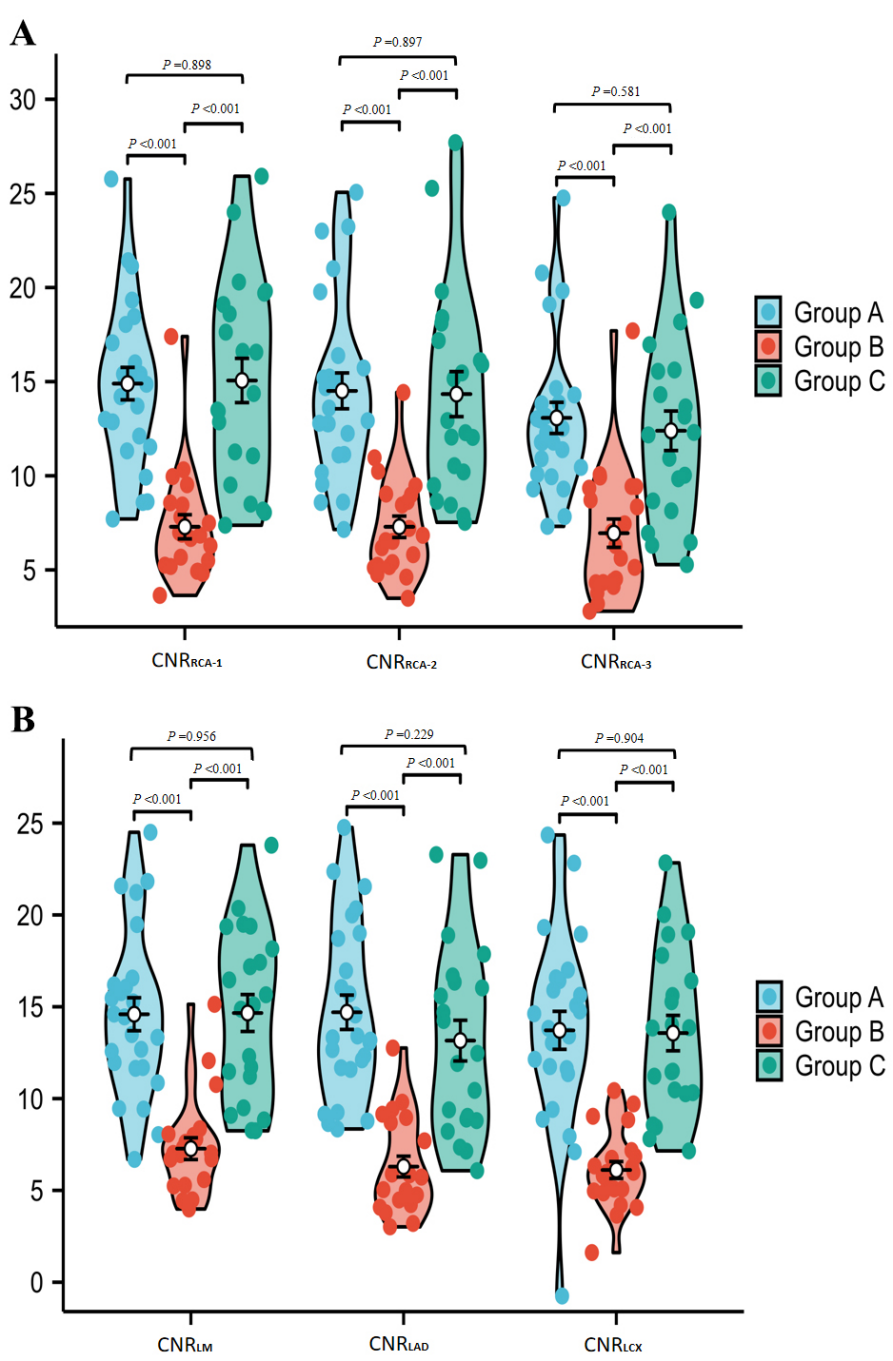
**Comparison of CNR in various vessels of the patient’s CCTA images**. (A) (RCA-1/2/3) and (B) (LM/LAD/LCX) display CNR measurements. In CNR analyses, Group A showed significantly higher CNRs than Group B in all measured coronary segments (*p* < 0.05). There was no statistically significant difference in CNR between Group A and Group C in any vessel. Moreover, Group C had substantially greater CNRs than Group B in all blood vessels (*p* < 0.05).

Group A had significantly higher CNRs than Group B in all blood vessels (*p* < 0.05). However, there was no significant difference in CNR between Group A and Group C in any blood vessel (*p* > 0.05). Group C had substantially greater CNRs in all blood vessels when compared to Group B (*p* < 0.05) Table 2. Fig. 7A,B).

#### 3.2.3 Effect Size Quantification

Effect sizes (partial η^2^) were calculated to quantify the magnitude of differences:

Large Effects (η^2^ > 0.14): CNR: RCA-1 (0.415), RCA-2 (0.365), LM (0.424), LAD (0.428), LCX (0.422). ◦ CT Values: RCA-1 (0.528), RCA-2 (0.494), LM (0.499), LAD (0.554), LCX (0.533). ◦ SD: RCA-2 (0.308), RCA-3 (0.282).

Medium Effects (0.06 ≤ η^2^ ≤ 0.14): ◦ SNR: RCA-2 (0.153), LAD (0.182), LCX (0.188). ◦ SD: LAD (0.126).

Small Effects (η^2^ < 0.06): ◦ SD: RCA-1 (0.001), LM (0.008), LCX (0.034).

#### 3.2.4 Paired Analysis Between Groups B and C

To address data dependency between Groups B and C, paired *t*-tests were performed. The results demonstrated significant improvements in image quality after applying CE-Boost and AiCE: CNR: All coronary segments exhibited significant improvement post CE-Boost (t = –4.576 to –7.225, *p*_adj_ < 0.05). SNR: Proximal vessels showed marked enhancement (t = –2.924 to –4.415, *p*_adj_ < 0.05). CT values: Vascular enhancement increased by 103.2–145.6 HU (t = –3.479 to –7.736, *p*_adj_ < 0.05). No significant noise difference was observed in most segments (t = –0.202 to 1.122, *p* = 0.275–0.992). These findings align with ANOVA results (**Supplementary Table 1**).

#### 3.2.5 Adjustment for Confounding Factors

Bonferroni-corrected multivariable regression adjusted for age and BMI demonstrated clinically critical insights: Group A exhibited superior vascular CT values (β: –0.24 to –0.11, 95% CI exclusive of null) compared to Group B (*P*1 < 0.05), while maintaining diagnostic parity with Group C (*P*2 = 0.320–0.888) in the LAD, LCX, LM, and RCA segments. The observed equivalence persisted despite triple-low dose reductions, with Group B/C comparisons exempted from adjustment due to inherent cohort homogeneity (age/BMI χ^2^ = 1.32, *p* = 0.251). These analyses confirm CE-Boost + AiCE reconstruction effectively counters vascular enhancement variability under low-dose conditions. Full statistical outcomes, including adjusted *P*1 (*A* vs. *B*) and *P*2 (*A* vs. *C*), are systematically cataloged in **Supplementary Table 2**.

In the results, large effects were primarily found in the CNR and CT values, suggesting significant improvements from CE-Boost and AiCE. Meanwhile, SD changes had minimal impact, with small or medium effect sizes, indicating protocol stability.

## 4. Discussion

CCTA is currently widely utilized, making the management of radiation dose and contrast agent dosage critical considerations. The primary objective of this study is to minimize radiation exposure and contrast agent-related complications while ensuring diagnostic image quality. We aim to introduce CE-Boost technology and deep learning reconstruction to achieve optimal diagnostic image quality under the triple-low CCTA protocol. Ultimately, the triple-low CCTA protocol employed in this study led to a significant reduction in overall contrast agent dosage by 67.8% and a decrease in effective radiation dose by 52.0% when compared to the conventional CCTA protocol. Nonetheless, the triple-low CCTA protocol presents certain challenges to image quality. The image quality of coronary arteries depends on the enhancement intensity of the coronary arteries and their contrast with surrounding soft tissues. Previous studies have demonstrated that the most direct way to improve the enhancement intensity of coronary arteries is to increase the dosage and iodine concentration of the contrast agent [9, 11, 17, 21], consistent with our findings. Although with the same iodine concentration, the images in Group B, which used a reduced contrast agent dosage, showed significantly lower CT values in all branch vessels compared to the images in Group A, which used a conventional dosage. In this study, Group C employed CE-Boost technology, which uses an accurate deformation registration algorithm based on non-contrast and contrast-enhanced images to further improve the CT value of enhanced CT images, thereby enhancing the image contrast [22]. Similar results were found in our study, showing that the CT values of Group C images using CE-Boost technology were significantly increased than those of Group B images without CE-Boost technology. Additionally, no increased artifacts were observed after applying CE-Boost technology. Our findings align with Xu *et al*. [11], who reported a 28% improvement in vascular contrast using CE-Boost in abdominal CTA, and Bernard *et al*. [16], who demonstrated a 40% radiation dose reduction with AiCE in cardiac imaging. However, unlike prior studies focusing on single-technique optimizations, our work integrates both technologies to address dual challenges of contrast and dose reduction.

Currently, dual-energy scanning and lowering tube voltage are commonly employed to enhance coronary lumen contrast while reducing the dosage of contrast agents in CCTA. However, dual-energy for CCTA can compromise temporal resolution, resulting in increased motion artifacts and potentially higher radiation doses. This outcome contradicts the original intent of our study design [23]. To mitigate the effects of reduced contrast agent dosage on CT values and address radiation dose concerns, we employed a tube voltage of 100 kV for scanning in this study. However, it is important to note that while images obtained using low kV techniques can produce high-quality vascular signals, they may also introduce noise and artifacts due to beam hardening [24]. In this study, the triple-low protocol implemented in Group B did not cause a significant increase in image noise; rather, it demonstrated a slight reduction. This enhancement can be attributed to the AiCE algorithm, which effectively mitigates noise from low-dose scanning and background noise associated with CE-Boost vascular enhancement, thereby fulfilling diagnostic requirements. The results indicate that 100 kV tube voltage may be appropriate. Although the CT values in Group B were relatively low, this did not result in a significant increase in image noise. Furthermore, the post-CE-Boost processing in Group C yielded CT values within a “reasonable” range for vascular lumens.

It is worth noting that preliminary research reports indicate that while CE-Boost can enhance image contrast, the multiple data overlays can lead to an accumulation of background noise, further increasing the background noise in the images [25]. To minimize the potential impact of CE-Boost and low-kV imaging on image noise, we utilized a novel reconstruction algorithm in this study—deep learning reconstruction (AiCE). AiCE reconstruction technology primarily leverages deep learning techniques to effectively reduce noise in signals [26, 27]. CE-Boost employs deformable registration algorithms to align non-contrast and contrast-enhanced phases, effectively isolating vascular enhancement while suppressing motion artifacts [10]. This approach synergizes with AiCE, a deep learning reconstruction framework trained on low-dose/high-dose image pairs, which discriminates anatomical structures from noise through hierarchical feature extraction [14, 16]. Together, these technologies compensate for the inherent trade-offs of the triple-low protocol. In our study, the images from Group C, which utilized AiCE reconstruction technology, displayed lower noise levels in all blood vessels except for the left main coronary artery when compared to those in Group A. Regarding diagnostic performance**,** there were no statistically significant differences in the CNRs between Group C images and Group A images obtained through the combined use of CE-Boost technology and AiCE reconstruction. The SNRs of the Group C images were significantly better than that of the Group B images. Compared with Group B, the enhanced CNR in Group C images led to a better evaluation of the degree of coronary artery stenosis.

There are certain limitations to this study. First, the sample size is relatively small, and future studies should incorporate larger sample sizes for more comprehensive analysis. Secondly, participants in Group A and Group B came from different cohorts in terms of radiation dose and contrast agent dosage. Lastly, our study lacks an evaluation of the clinical diagnostic efficacy of the three groups. Future studies should correlate triple-low CCTA findings with invasive angiography to validate diagnostic accuracy, and assess adaptability in arrhythmic populations.

## 5. Conclusion

This study indicates that compared to conventional CCTA, the triple-low CCTA protocol using CE-Boost technology combined with AiCE reconstruction technology provides comparable image quality. Therefore, these results propose that triple-low CCTA using CE-Boost technology combined with AiCE reconstruction technology can be clinically applied, especially in the screening of coronary artery disease.

## Availability of Data and Materials

The datasets used and/or analysed during the current study are available from the corresponding author on reasonable request.

## Supplementary Material

Supplementary material associated with this article can be found, in the online version, at https://doi.org/10.31083/RCM31334.

## References

[1] Dziedzic EA, Gąsior JS, Tuzimek A, Dąbrowski M, Jankowski P (2022). Neutrophil-to-Lymphocyte Ratio Is Not Associated with Severity of Coronary Artery Disease and Is Not Correlated with Vitamin D Level in Patients with a History of an Acute Coronary Syndrome. *Biology*.

[2] Ding J, Wu J, Wei H, Li S, Huang M, Wang Y (2022). Exploring the Mechanism of Hawthorn Leaves Against Coronary Heart Disease Using Network Pharmacology and Molecular Docking. *Frontiers in Cardiovascular Medicine*.

[3] Wang B, Kou W, Ji S, Shen R, Ji H, Zhuang J (2022). Prognostic value of plasma adipokine chemerin in patients with coronary artery disease. *Frontiers in Cardiovascular Medicine*.

[4] Litmeier S, Meinel TR, von Rennenberg R, Kniepert JU, Audebert HJ, Endres M (2022). Coronary angiography in acute ischemic stroke patients: frequency and determinants of pathological findings in a multicenter cohort study. *Journal of Neurology*.

[5] Wu L, Huang G, Yu X, Ye M, Liu L, Ling Y (2022). Deep Learning Networks Accurately Detect ST-Segment Elevation Myocardial Infarction and Culprit Vessel. *Frontiers in Cardiovascular Medicine*.

[6] Kim YD, Kim YK, Yoon YE, Yoon CH, Park KH, Woo SJ (2019). Association of Retinal Artery Occlusion with Subclinical Coronary Artery Disease. *Journal of Korean Medical Science*.

[7] Katerji M, Bertucci A, Filippov V, Vazquez M, Chen X, Duerksen-Hughes PJ (2022). Proton-induced DNA damage promotes integration of foreign plasmid DNA into human genome. *Frontiers in Oncology*.

[8] Li M, Li L, Qin Y, Luo E, Wang D, Qiao Y (2022). Elevated TyG Index Predicts Incidence of Contrast-Induced Nephropathy: A Retrospective Cohort Study in NSTE-ACS Patients Implanted With DESs. *Frontiers in Endocrinology*.

[9] Stocker TJ, Leipsic J, Hadamitzky M, Chen MY, Rubinshtein R, Deseive S (2020). Application of Low Tube Potentials in CCTA: Results From the PROTECTION VI Study. *JACC. Cardiovascular Imaging*.

[10] Baerends E, Oostveen LJ, Smit CT, Das M, Sechopoulos I, Brink M (2018). Comparing dual energy CT and subtraction CT on a phantom: which one provides the best contrast in iodine maps for sub-centimetre details?. *European Radiology*.

[11] Xu J, Wang S, Wang X, Wang Y, Xue H, Yan J (2022). Effects of contrast enhancement boost postprocessing technique in combination with different reconstruction algorithms on the image quality of abdominal CT angiography. *European Journal of Radiology*.

[12] Lu Y, Cao R, Jiao S, Li L, Liu C, Hu H (2024). A novel method of carotid artery wall imaging: black-blood CT. *European Radiology*.

[13] Hou J, Zhang Y, Yan J, Zhang T, Xia W, Zhu Y (2023). Clinical application of the contrast-enhancement boost technique in computed tomography angiography of the portal vein. *Abdominal Radiology (New York)*.

[14] Chen H, Zhang Y, Zhang W, Liao P, Li K, Zhou J (2017). Low-dose CT via convolutional neural network. *Biomedical Optics Express*.

[15] Cai H, Jiang H, Xie D, Lai Z, Wu J, Chen M (2024). Enhancing image quality in computed tomography angiography follow-ups after endovascular aneurysm repair: a comparative study of reconstruction techniques. *BMC Medical Imaging*.

[16] Bernard A, Comby PO, Lemogne B, Haioun K, Ricolfi F, Chevallier O (2021). Deep learning reconstruction versus iterative reconstruction for cardiac CT angiography in a stroke imaging protocol: reduced radiation dose and improved image quality. *Quantitative Imaging in Medicine and Surgery*.

[17] Abbara S, Blanke P, Maroules CD, Cheezum M, Choi AD, Han BK (2016). SCCT guidelines for the performance and acquisition of coronary computed tomographic angiography: A report of the society of Cardiovascular Computed Tomography Guidelines Committee: Endorsed by the North American Society for Cardiovascular Imaging (NASCI). *Journal of Cardiovascular Computed Tomography*.

[18] Leipsic J, Abbara S, Achenbach S, Cury R, Earls JP, Mancini GJ (2014). SCCT guidelines for the interpretation and reporting of coronary CT angiography: a report of the Society of Cardiovascular Computed Tomography Guidelines Committee. *Journal of Cardiovascular Computed Tomography*.

[19] Schönfeld T, Seitz P, Krieghoff C, Ponorac S, Wötzel A, Olthoff S (2024). High-pitch CT pulmonary angiography (CTPA) with ultra-low contrast medium volume for the detection of pulmonary embolism: a comparison with standard CTPA. *European Radiology*.

[20] Chatzaraki V, Kubik-Huch RA, Thali M, Niemann T (2022). Quantifying image quality in chest computed tomography angiography: Evaluation of different contrast-to-noise ratio measurement methods. *Acta Radiologica (Stockholm, Sweden: 1987)*.

[21] Xing Y, Azati G, Pan CX, Dang J, Jha S, Liu WY (2015). Improving Patient to Patient CT Value Uniformity with an Individualized Contrast Medium Protocol Tailored to Body Weight and Contrast Medium Concentration in Coronary CT Angiography. *PloS One*.

[22] Otgonbaatar C, Jeon PH, Ryu JK, Shim H, Jeon SH, Ko SM (2023). The effectiveness of post-processing head and neck CT angiography using contrast enhancement boost technique. *PloS One*.

[23] Tarkowski P, Czekajska-Chehab E (2021). Dual-Energy Heart CT: Beyond Better Angiography-Review. *Journal of Clinical Medicine*.

[24] Cha MJ, Kim SM, Ahn TR, Choe YH (2020). Comparing feasibility of low-tube-voltage protocol with low-iodine-concentration contrast and high-tube-voltage protocol with high-iodine-concentration contrast in coronary computed tomography angiography. *PloS One*.

[25] Li J, Zhang Y, Hou J, Li Y, Zhao Z, Xu M (2024). Clinical Application of Dark-blood Imaging in Head and Neck CT Angiography: Effect on Image Quality and Plaque Visibility. *Academic Radiology*.

[26] Greffier J, Dabli D, Hamard A, Belaouni A, Akessoul P, Frandon J (2022). Effect of a new deep learning image reconstruction algorithm for abdominal computed tomography imaging on image quality and dose reduction compared with two iterative reconstruction algorithms: a phantom study. *Quantitative Imaging in Medicine and Surgery*.

[27] Nakamura Y, Higaki T, Tatsugami F, Honda Y, Narita K, Akagi M (2020). Possibility of Deep Learning in Medical Imaging Focusing Improvement of Computed Tomography Image Quality. *Journal of Computer Assisted Tomography*.

