# Supplementary material for: Feasibility Study of Triple-low CCTA for Coronary Artery Disease Screening Combining Contrast Enhancement Boost and Deep Learning Reconstruction
Source: Rev Cardiovasc Med. 2025 Jun 30;26(6):31334. doi: 10.31083/RCM31334 (PMC12230822; doi:10.31083/RCM31334)
Supplement: Supplementary file 1 [file 2153-8174-26-6-31334-s1.zip › Supplementary Material.docx]

| **Image parameters** | | **t** | ***P***_adj_ |
| --- | --- | --- | --- |
| CT value | CT_LM_ | -5.832 | ＜0.001 |
|  | CT_LAD_ | -4.899 | ＜0.001 |
|  | CT_LCX_ | -7.736 | ＜0.001 |
|  | CT_RCA-1_ | -5.602 | ＜0.001 |
|  | CT_RCA-2_ | -6.242 | ＜0.001 |
|  | CT_RCA-3_ | -3.479 | 0.002 |
| SD | SD_LM_ | 0.010 | 0.992 |
|  | SD_LAD_ | 0.230 | 0.821 |
|  | SD_LCX_ | 1.122 | 0.275 |
|  | SD_RCA-1_ | -0.202 | 0.842 |
|  | SD_RCA-2_ | 1.023 | 0.319 |
|  | SD_RCA-3_ | 0.558 | 0.583 |
| SNR | SNR_LM_ | -4.415 | ＜0.001 |
|  | SNR_LAD_ | -3.622 | 0.002 |
|  | SNR_LCX_ | -3.427 | 0.003 |
|  | SNR_RCA-1_ | -2.924 | 0.008 |
|  | SNR_RCA-2_ | -3.251 | 0.004 |
|  | SNR_RCA-3_ | -3.871 | 0.001 |
| CNR | CNR_LM_ | -6.222 | ＜0.001 |
|  | CNR_LAD_ | -5.818 | ＜0.001 |
|  | CNR_LCX_ | -7.225 | ＜0.001 |
|  | CNR_RCA-1_ | -5.890 | ＜0.001 |
|  | CNR_RCA-2_ | -5.689 | ＜0.001 |
|  | CNR_RCA-3_ | -4.576 | ＜0.001 |

Supplementary Table 1. Paired *t*-test results between Groups B and C.

*P*_adj_ values are calculated using Bonferroni correction and are used to report the significance of the intergroup comparisons. *P*_adj_ < 0.05 indicates statistical significance after multiple comparison correction.

| **Image parameters** | | ***P1*** | ***P2*** |
| --- | --- | --- | --- |
| CT value | CT_LM_ | 0.001 | 0.002 |
|  | CT_LAD_ | 0.034 | 0.002 |
|  | CT_LCX_ | 0.001 | 0.003 |
|  | CT_RCA-1_ | 0.002 | 0.003 |
|  | CT_RCA-2_ | 0.012 | 0.002 |
|  | CT_RCA-3_ | 0.001 | 0.001 |
| SD | SD_LM_ | 0.323 | 0.299 |
|  | SD_LAD_ | 0.058 | 0.049 |
|  | SD_LCX_ | 0.430 | 0.145 |
|  | SD_RCA-1_ | 0.921 | 0.892 |
|  | SD_RCA-2_ | 0.006 | 0.005 |
|  | SD_RCA-3_ | 0.007 | 0.009 |
| SNR | SNR_LM_ | 0.001 | 0.055 |
|  | SNR_LAD_ | 0.006 | 0.320 |
|  | SNR_LCX_ | 0.004 | 0.329 |
|  | SNR_RCA-1_ | 0.001 | 0.018 |
|  | SNR_RCA-2_ | 0.341 | 0.077 |
|  | SNR_RCA-3_ | 0.873 | 0.036 |
| CNR | CNR_LM_ | 0.001 | 0.856 |
|  | CNR_LAD_ | 0.003 | 0.407 |
|  | CNR_LCX_ | 0.001 | 0.933 |
|  | CNR_RCA-1_ | 0.001 | 0.741 |
|  | CNR_RCA-2_ | 0.001 | 0.888 |
|  | CNR_RCA-3_ | 0.001 | 0.742 |

Supplementary Table 2. Age- and BMI-adjusted *p*-values for comparisons of CT value, SD, SNR, and CNR between Groups A, B, and C.

P1 ：The age and BMI adjusted P-value for the comparison between Group A and Group B.

P2 ：The age and BMI adjusted P-value for the comparison between Group A and Group C.

**Supplementary Fig. 1. Visual Reference for 5-Point Image Quality Scoring System.**


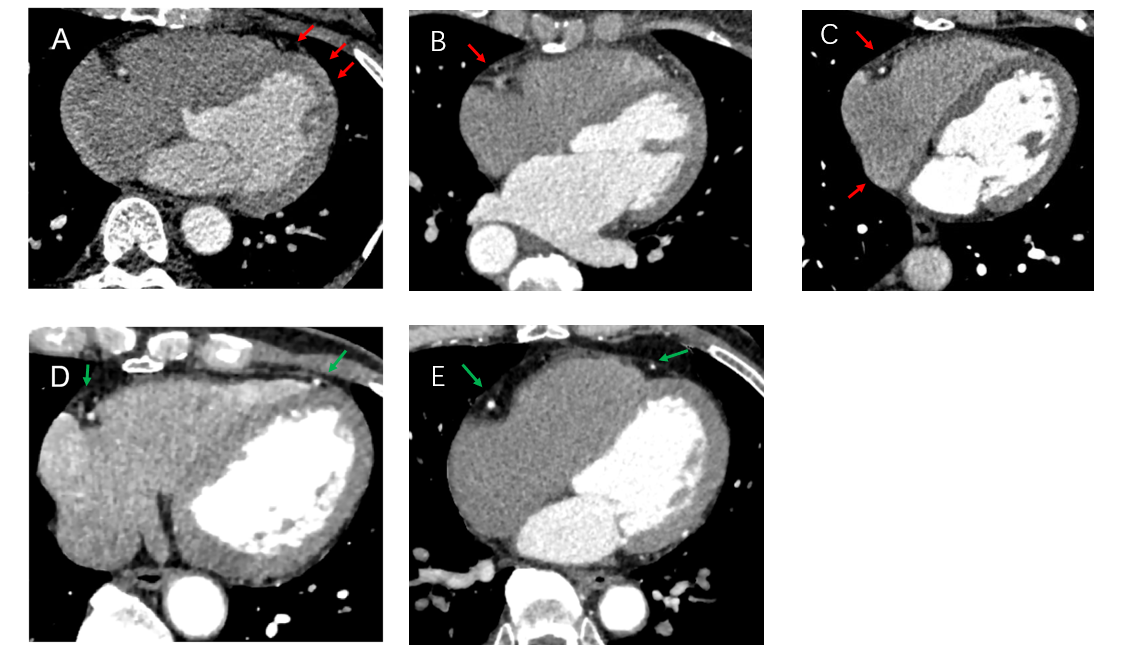
 (A) Score 1: Non-diagnostic due to excessive noise and blurred LAD margins (red arrows). (B) Score 2: Severe noise obscuring RCA (red arrows). (C) Score 3: moderate noise obscuring vascular margins, clear vascular reconstruction, and slightly unclear display of small anatomical structures (red arrows). (D) Score 4: Mild noise but preserved small branch visibility (green arrows). (E) Score 5: Excellent image quality with sharp vascular boundaries (green arrows) and no discernible noise.
