# Supplementary figures and images for: Feasibility Study of Triple-low CCTA for Coronary Artery Disease Screening Combining Contrast Enhancement Boost and Deep Learning Reconstruction
Source: Rev Cardiovasc Med. 2025 Jun 30;26(6):31334. doi: 10.31083/RCM31334 (PMC12230822; doi:10.31083/RCM31334)

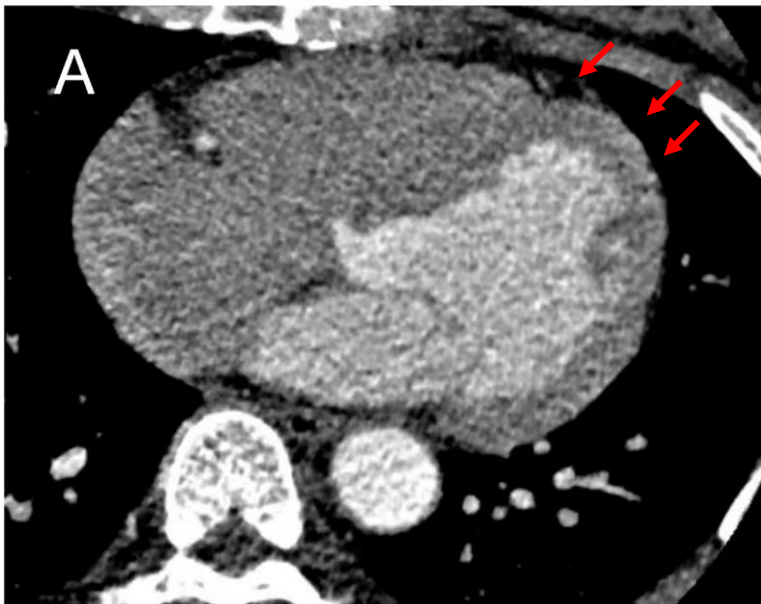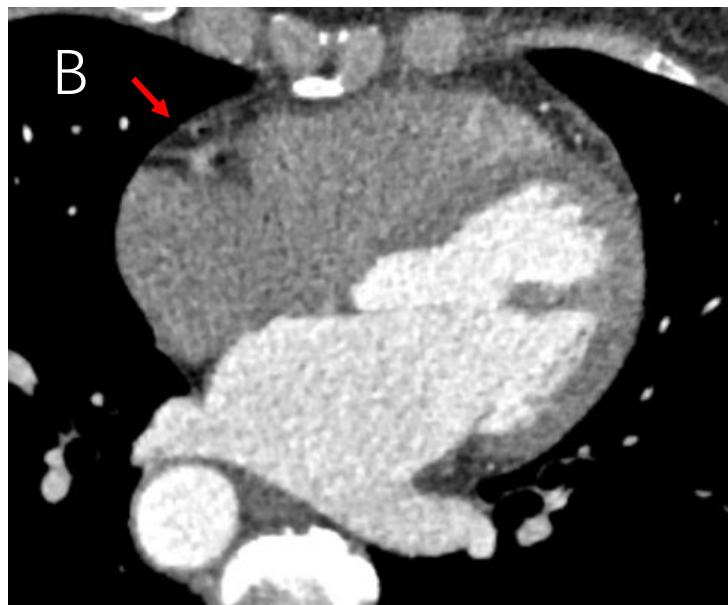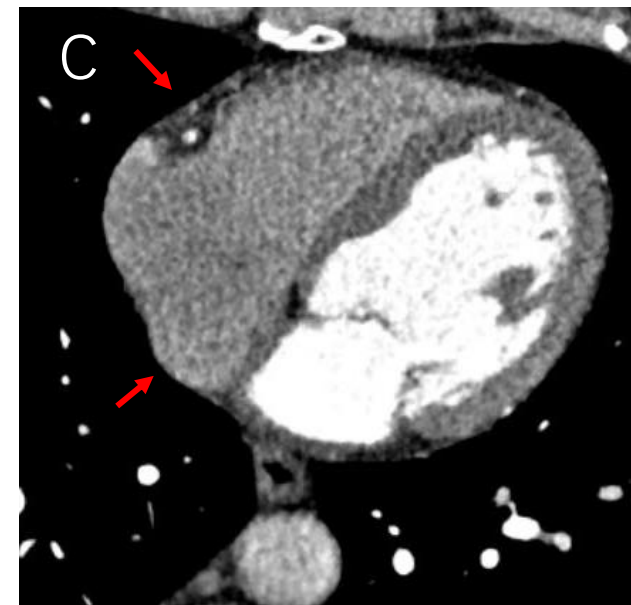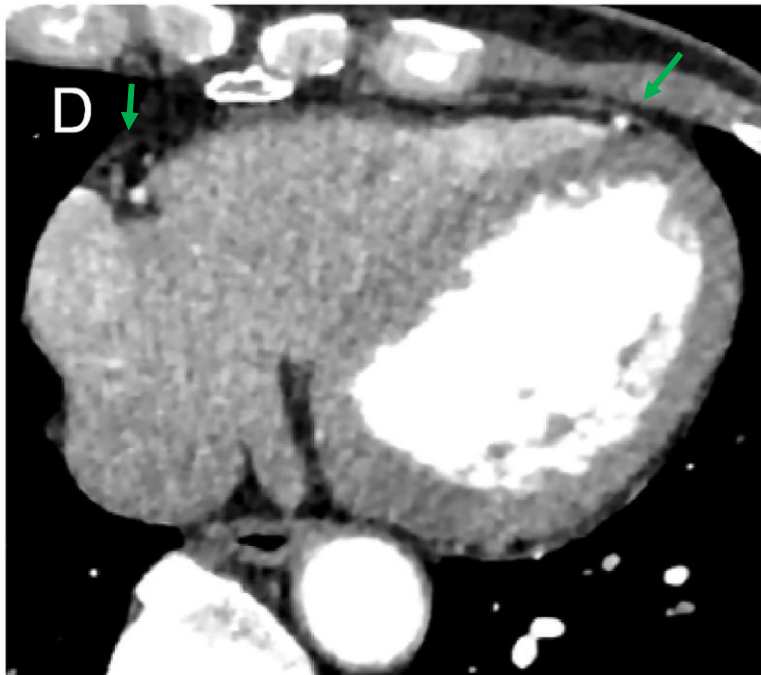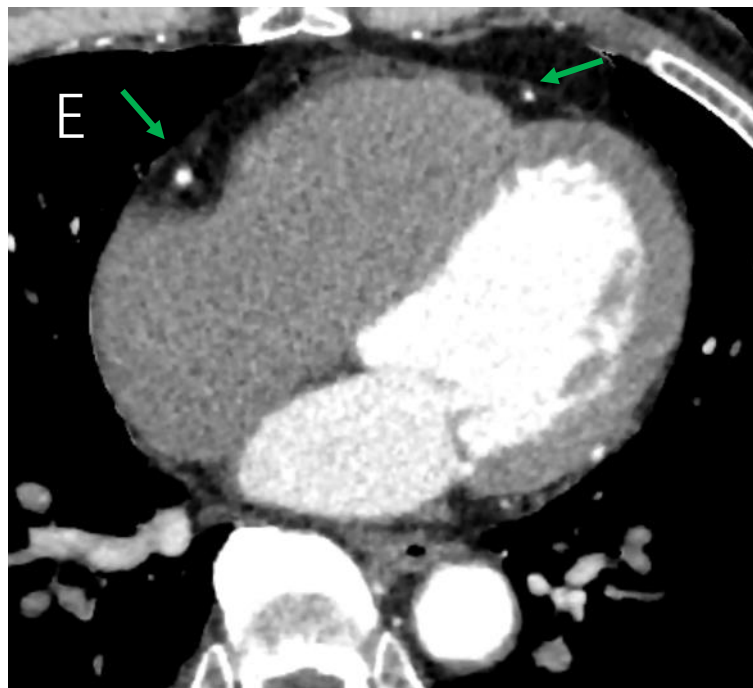

Supplement: Supplementary file 1 [file 2153-8174-26-6-31334-s1.zip › Supplementary Fig.1 .pdf]
